# The impacts of resveratrol on the retinal degeneration in a rat model of retinitis pigmentosa induced by alkylation: an in-vivo study

**DOI:** 10.1080/19768354.2023.2226695

**Published:** 2023-06-26

**Authors:** Weiming Yan, Yan Sun, Yutong Wang, Wangjiao Liang, Yuxin Xia, Weihua Yan, Meizhu Chen, Tao Chen, Dongliang Li

**Affiliations:** a Department of Ophthalmology, The 900th Hospital of Joint Logistic Support Force, PLA (Clinical Medical College of Fujian Medical University, Dongfang Hopsital Affiliated to Xiamen University), Fuzhou, People’s Republic of China; b Center of Clinical Aerospace Medicine, Air Force Military Medical University, Xi’an, People’s Republic of China; c Changtai No.2 High School of Fujian Province, Zhangzhou, People’s Republic of China; d Department of Hepatobiliary Disease, The 900th Hospital of Joint Logistic Support Force, PLA, Fuzhou, People’s Republic of China

**Keywords:** Retinitis pigmentosa, resveratrol, alkylation, electroretinogram, SIRT1

## Abstract

Upregulation of Sirtuin Type 1 (SIRT1), a nicotinamide adeninedinucleotide (NAD^+^)-dependent deacetylase, has been proved to protect against ample ocular diseases, while its effect on retinitis pigmentosa (RP) has not been illustrated. The study was aimed to explore the impacts of resveratrol (RSV), a SIRT1 activator, on the photoreceptor degeneration in a rat model of RP induced by N-methyl-N-nitrosourea (MNU), an alkylation. The rats were induced RP phenotypes via the intraperitoneal injection of MNU. The electroretinogram was conducted and revealed that RSV could not prevent the decline of retinal function in the RP rats. The optical coherence tomography (OCT) and the retinal histological examination were performed and showed that the reduced thickness of the outer nuclear layer (ONL) was not preserved by RSV intervention. The immunostaining technique was applied. Afther the MNU administration, the number of the apoptotic photoreceptors in the ONL throughout the retinasand the number of microglia cells present among the outer part throughout the retinas were not significantly reduced by RSV. Western blotting was also performed. The data showed that the level of SIRT1 protein was decreased after MNU administration, while RSV was not able to obviously alleviate the downregulation. Our data together indicated that RSV was not able to rescue the photoreceptor degeneration in the MNU-induced RP rats, which might be due to the MNU-induced consumption of the NAD^+^.

## Introduction

Retinitis pigmentosa (RP), a prevalent retinal disease characterized by the loss of photoreceptors, would lead to an obvious change of fundus appearance and deterioration of retinal function (Kelbsch et al. 2017). Although the gene therapy has shown a great potential in ameliorating the retinal degeneration (Fischer et al. 2017), the overall outcome is far from satisfactory due to the high heterogeneity of RP. Effective medicine to mitigate or rescue the photoreceptor death is continuously required in urgent need (Martin-Merida et al. 2017).

While the detailed molecular mechanism for the RP pathogenesis remains unclear, the loss of photoreceptors is commonly attributed to the apoptosis (Kang and Yu 2017). Sirtuin Type 1 (SIRT1), an NAD^+^-dependent deacetylase, is a key role in regulating apoptosis (Kumar and Chauhan 2016). It could render an anti-apoptotic effect by regulating the activity of the apoptosis proteins such as BAX, Bcl-2 and Caspase-3 through a reversible deacetylation (Tang 2016). Besides, the anti-oxidative effect of SIRT1 through upregulating the expression of antioxidant enzymes could also contribute to ameliorating the apoptosis (Chan et al. 2018). In retinas, SIRT1 was reported to be extensively expressed (Mimura et al. 2013). Resveratrol (RSV), a polyphenolic SIRT1-activating compound firstly identified by Wood et al. (Wood et al. 2004), is beneficial for the relief of multiple diseases (Banu et al. 2016; Fenner 2017). The SIRT1 activation by RSV has been demonstrated to alleviate the photoreceptor damage in retinal diseases, such as the age-related macular degeneration (AMD) and the diabetic retinopathy (DR) (Nagineni et al. 2014; Petrovic 2014). Besides, RSV was confirmed to protect photoreceptor cells through inhibition of apoptosis and oxidative stress in the rodent models of light-induced retinopathy (Qi et al. 2015).

Based on the aforementioned evidence, SIRT1 is deemed to serve as an attractive target for ameliorating retinal photoreceptor apoptosis. However, whether the SIRT1 activation could alleviate RP has not been elucidated. During the current research, we evaluated the impacts of RSV, the SIRT1 activator, on the photoreceptor degeneration in a rat model of RP in order to explore new protective measures against RP.

## Materials and methods

### Animals and RP modeling

The adult male Sprague–Dawley (SD) rats (aged 8–9 weeks old) were purchased from the Laboratory Animal Center of the Air Force Military Medical University (License no.: #2014270138S). The rats handling and the experimental protocols were abided by the Association for Research in Vision and Ophthalmology Statement (ARVO) for ophthalmic use and were approved by the Animal Care and Use Committee of the 900th Hospital of Chinese PLA and the Air Force Military Medical University. In total, seventy-two rats were averagely delivered into the Norm (N) group, the Model (M) group and the resveratrol treatment (R) group randomly. N-methyl-N-nitrosourea (MNU, Lot#F1518137, Aladdin, Shanghai, China), an akylation, was kept at 4°C before use, and was then dissolved in saline containing 0.05% acetic acid. Rats from the M and the R groups were intraperitoneally injected with MNU at 50 mg/kg to induce the RP model as previously reported (Yan et al. 2020).

### RSV administration and experimental design

RSV (Lot#70675, Cayman Chemical, US) was dissolved in the dimethyl sulfoxide (DMSO) at 65 mg/ml and then diluted with phosphate buffer saline (PBS) to get a 6.67% concentration of DMSO (Qi et al. 2015). RSV was administered to the rats of the R group by gavage at two modes: (1) a dose of 200 mg/kg at 0 h (h), 2, 10 h after the MNU-administration, and then once a day for the following two days; (2) a daily dose of 50 mg/kg 3 days (d) prior to and 3 d after the MNU-administration. The rats of the M group were administered with the same volume of vehicle by gavage at the same time.

Rats were examined at the time point of 1 and 3 d after the MNU administration. Electroretinogram (ERG) was conducted to assess the retinal function, with fundus photography, optical coherence tomography (OCT) and fundus fluorescence angiography (FFA) performed to examine the morphology of the retinas. After that, the rat eyes were harvested for retinal histological examination, terminal deoxyuridine triphosphate nick-end labeling (TUNEL) analysis and immunofluorescence staining. Besides, the rat retinas were subjected to protein studies by Western blotting.

### ERG recording

The rats were subjected to an overnight dark adaption (>12 h) and then were deeply anesthetized as previously described (Yan et al. 2020). Briefly, the pupils of the rats were dilated. The center of the rat cornea was attached with a ring electrode that served as the active electrode. Meanwhile, the reference electrode and the ground electrode were inserted beneath the skin and the tail, respectively. ERG responses according to the guidelines of the International Society for Clinical Electrophysiology of Vision (ISCEV) were recorded, using the Full-field stimulator (Ganzfeld) and a computer system (RETI port; Roland, Germany) (McCulloch et al. 2015). Afterwards, the Levofloxacin Eye Drops (Lot#20103148, Suzhou, China) were applied on the rat corneas to avoid infection.

### Fundus photography, OCT scanning and FFA examinations

After the ERG recording, the pupils of the rats were kept dilated. The Retinal Imaging System and 4D-ISOCT Microscope Imaging System (OptoProbe, Canada) were applied for the fundus, FFA imaging, and OCT scanning according to the instructions. Specifically, the position of the rat cornea was adjusted to center the optic disc on the screen to get the fundus and OCT images. After that, FFA was performed with the tail intravenous injection of the Sodium Fluorescein (Lot# H44023401, Guangzhou, China) at 10 ml/kg. The structure of the retinal vessels was imaged with the optic disc centered. The length between the inner border of outer nuclear layer (ONL) and the retinal pigment epithelium (RPE) 800 μm away from the optic disc at both sides was measured by the OCT Image Analysis Software (Version 2.0, Optoprobe, Canada).

### Retinal histological examination

The rat eyes were enucleated rapidly after injection of a lethal dose of pentobarbital. The eyecups were harvested and fixed in 4% paraformaldehyde (Mediatech, Inc., Herndon, VA, US) for 24 h. The tissues were dehydrated by the graded ethanols, and then paraffin-embedded. Serial sections of 4 μm in thickness were cut vertically through the optic disc. For each eye, 3 retinal sections were stained with hematoxylin and eosin (HE). The images were taken using a digital imaging system (DP71, Olympus, Japan). Analysis was conducted by counting the number of ONL rows at 200 μm (the middle region, MI), 2000 μm (the midperipheral region, MP) and 4000 μm (the peripheral region, P) away from the optic disc at the high magnification (×400).

### TUNEL analysis

Apoptosis was detected with the cell death detection POD Kit (Lot#11684795910, Roche Diagnostics GmbH, Mannheim, Germany). Briefly, the rat retinal sections were prepared for TUNEL assay according to the instruction. The sections were counterstained with DAPI (#C1005, Beyotime, Shanghai, China) and then analyzed under the digital immunofluorescence microscope imaging system (DP71, Olympus, Japan). The number of TUNEL-positive cells among the ONL was counted in 3 sections from each rat retina and was averaged.

### Immunostaining

The rat retinal sections were subjected for immunostaining of anti-ionized calcium-binding adapter molecule 1 (Iba1). Briefly, the sections was dehydrated and then mounted with 10% goat serum (Lot#121314B09, Boster, Wuhan, China) at room temperature for 1 h. Primary antibody against Iba1 (LOt#013-26471, 100 μg/100 μl, Host species-Rabbit, Wako, Japan) at 1:500 dilution were applied overnight at 4°C. Later, the secondary antibody (Lot#ZF-0516, 100 μg/100 μl, HRP-conjugated Goat Anti-Rabbit IgG, Beijing ZSGB-BIO, China) was applied on the sections for 1 h at 37°C. The retinal sections were then counterstained with DAPI. The middle (MI), midperipheral (MP) and peripheral (P) regions of the retinas were imaged by the fluorescence microscope (DP71, Olympus, Japan). The number of Iba1-positive cells was counted in 3 sections from each retina and was averaged under high magnification (×400).

### Western blotting

The rat retinas were harvested and then homogenized in RIPA buffer (Beyotime). 30 μg of the homogenized protein was subjected for electrophoresing, and was then incubated at 4°C with primary antibodies against SIRT1 (Lot#ab110304, 100 μg/100 μl, Host species-Mouse, Abcam, US) and β-actin (Lot#NC011, 100 μg/100 μl, Host species-Rabbit, Zhuangzhi, China) or Gapdh (Lot#10494-1-AP, 60 μg/100 μl, Host species-Rabbit, Proteintech, US) at 1:1,000 dilution overnight. The secondary antibodies (Lot#EK020, 100 μg/100 μl, HRP-conjugated Goat Anti-rabbit IgG, Zhuangzhi, China, or Lot#D110087-0100, 100 μg/100 μl, HRP-conjugated Goat Anti-Mouse IgG, Sangon Biotech, China) at 1:10,000 dilution were applied on the membranes for 1 h at room temperature. The protein bands among the membranes were detected by an enhanced chemiluminescence system (Thermo Fisher Scientific, US). The ImageJ software (Bethesda, MD, US) was applied to analyze the intensity of the bands.

### Statistical analysis

The data were expressed as mean ± standard error (S.E.). One-way analysis of variance (ANOVA) with multiple contrast analysis by Bonferroni test was performed via the SPSS software (version 16.0, Chicago, US). The difference was considered statistically significant when the *P* value was less than 0.05.

## Results

### The effect of RSV on retinal function of MNU-administered rats

**Figure 1.**
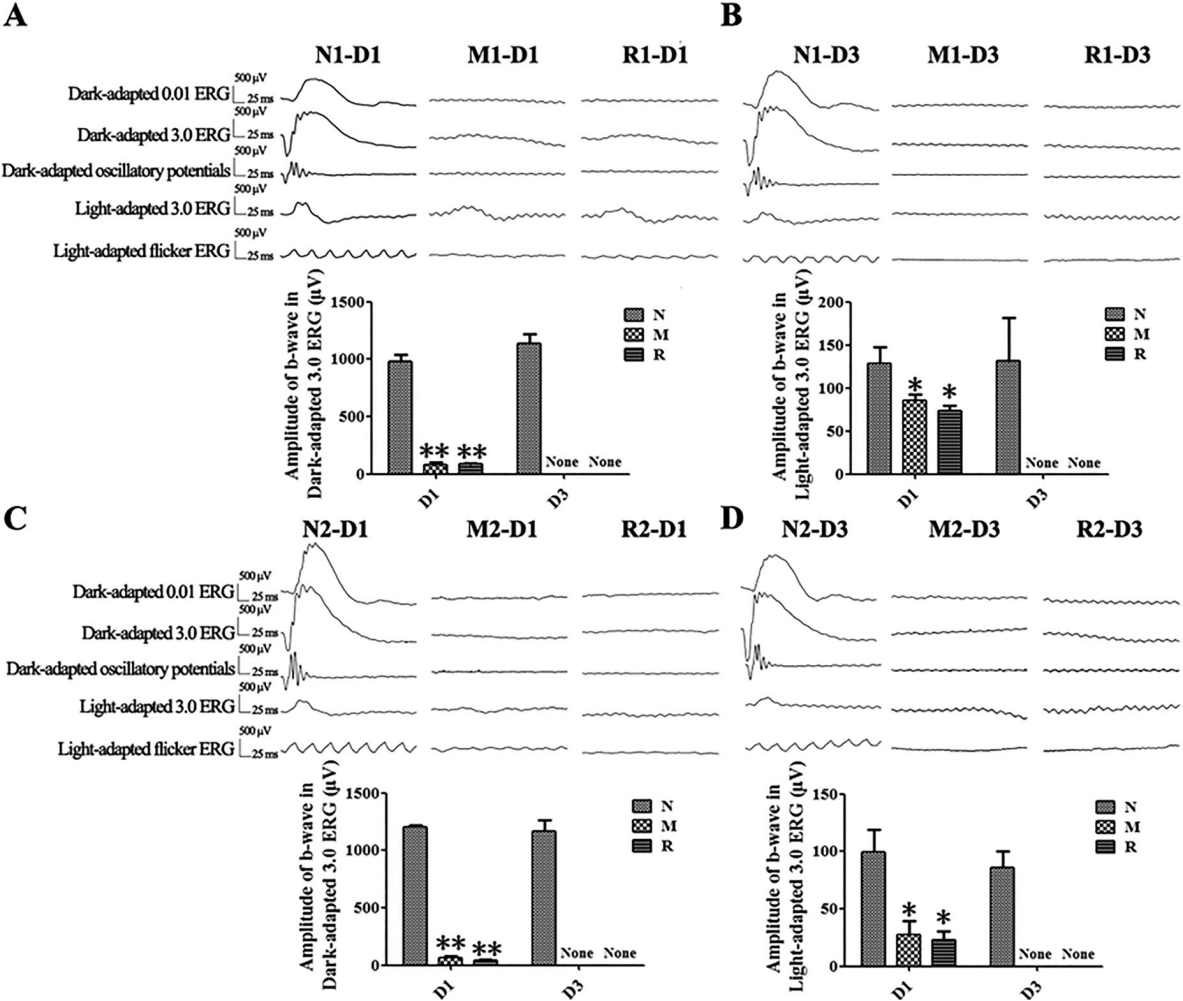
RSV-induced effect on retinal function in MNU-administered rats. A, B: Representative waveforms and plot of the b-wave amplitudes of Dark-adapted and Light-adapted ERG of rats with RSV intervention of the first mode at 1 d (A) and 3 d (B) after MNU administration; C, D: Representative waveforms and plot of the b-wave amplitudes of Dark-adapted and Light-adapted 3.0 ERG with RSV intervention of the second mode at 1 d (C) and 3 d (D) after MNU administration. (*n* = 3; *P < 0.05, **P < 0.01: vs. N group; None: no detection).

Compared to the N group, the b-wave amplitudes of the Dark-adapted 3.0 ERG and the Light-adapted 3.0 ERG from rats in the M group were decreased dramatically at 1 d and were extinguished at 3 d after the MNU injection (all *P* < 0.01). The b-wave amplitudes of rats in the R group did not show any significant differences from those of the M group at both 1 d (the M group vs. R group in the first mode: 82.75 ± 21.22 *vs.* 85.8 ± 7.57 μV for Dark-adapted 3.0 ERG, 85.25 ± 7.09 *vs.* 74.00 ± 5.86 μV for Light-adapted 3.0 ERG; the M group *vs.* R group in the second mode: 67.33 ± 12.71 *vs.* 44.33 ± 2.33 μV for Dark-adapted 3.0 ERG, 27.33 ± 11.86 *vs.* 22.67 ± 7.84 μV for Light-adapted3.0 ERG) and 3 d (all *P* > 0.05) (Figure 1).

### The effect of RSV on the retinal architecture after MNU damage

**Figure 2.**
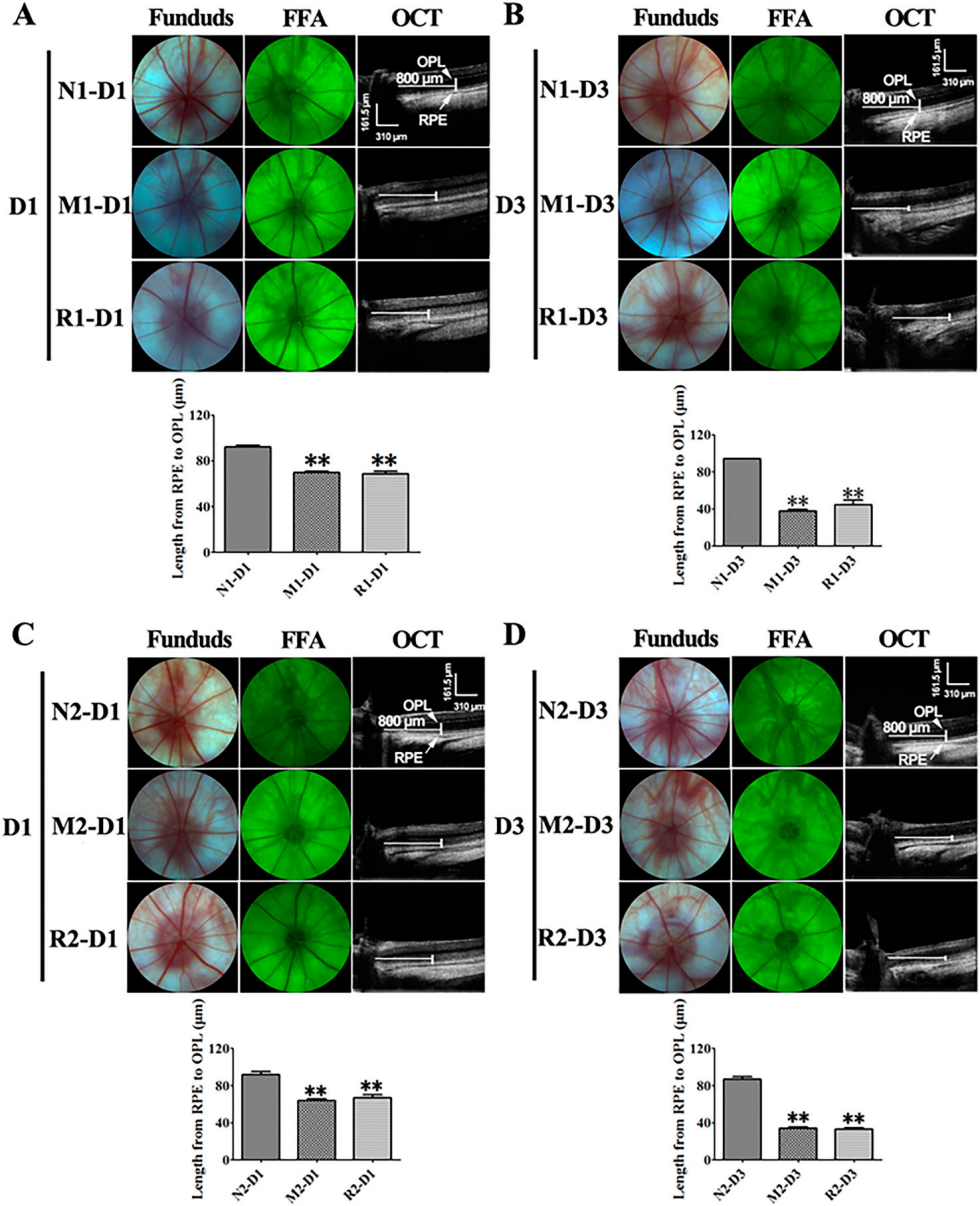
RSV-induced effect on living ocular structure of MNU-administrated rats. A, B: Typical images of fundus photograph, FFA, OCT of rats with RSV intervention of the first mode, and plot of length from junction of OPL and ONL to RPE at 1 d (A) and 3 d (B) after MNU administration; C, D: Typical images of fundus photograph, FFA, OCT of rats with RSV intervention of the second mode, and plot of length from junction of OPL and ONL to RPE at 1 d (A) and 3 d (B) after MNU administration. (*n* = 3; MI: the middle area of the retina; MP: the mid-peripheral area of the retina; P: the peripheral area of the retina; **P < 0.01: vs. N group).

The ONL of rat retinas turned gray and could not be easily distinguished from the outer plexiform layer (OPL) and the outer/inner segment on OCT image at 3 d after the MNU injection. The length between the inner border of ONL and the RPE was markedly reduced in the rat retinas of the M group in comparison to that of the N group at both 1 and 3 d (all *P* < 0.01). However, the data in the R group was not significantly different from that of the M group (all *P* > 0.05). No obvious changes were found in the morphology of the optic disc, retinal vessels by the fundus photography and FFA in all groups (Figure 2).

**Figure 3.**
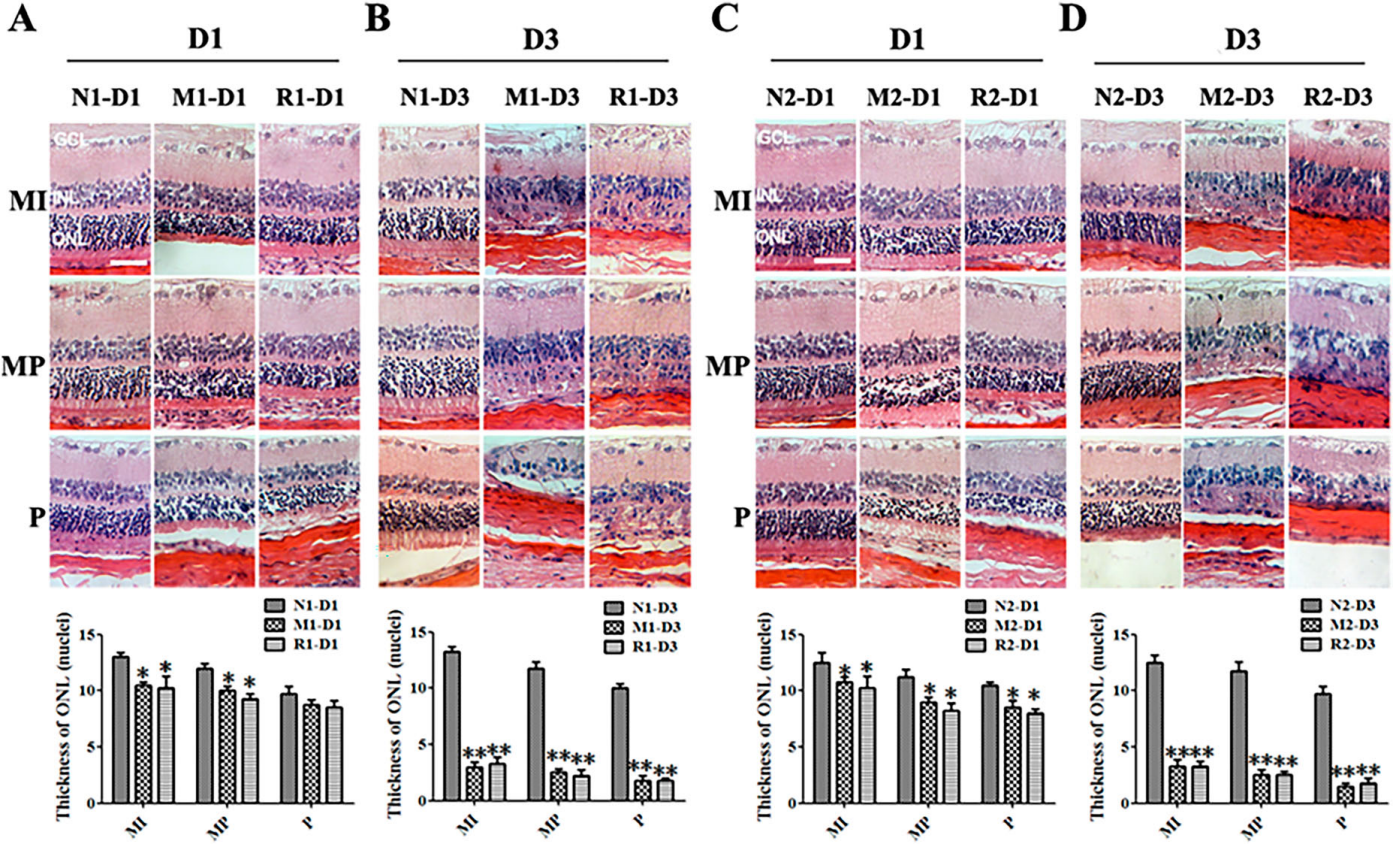
RSV-induced effect on retinal histology in MNU-treated retinas. A, B: Typical images of HE staining of retinal sections of rats with RSV intervention of the first mode and plot of nuclei counts of ONL layer at 1 d (A) and 3 d (B) after MNU administration; A, B: Typical images of HE staining of retinal sections of rats with RSV intervention of the first mode and plot of nuclei counts of ONL layer at 1 d (A) and 3 d (B) after MNU administration. C, D: Typical images of HE staining of retinal sections of rats with REV intervention of the second mode and plot of nuclei counts of ONL layer at1 d (A) and 3 d (B) after MNU administration. (*n* = 3; MI: the middle area of the retina; MP: the mid-peripheral area of the retina; P: the peripheral area of the retina; ONL: outer nuclear layer; INL: inner nuclear layer; GCL: ganglion cell layer; Scale: 50 μm; *P < 0.05, **P < 0.01: vs. N group).

In the HE staining of retinal sections, the nuclei counts of the ONL in the M group were significantly reduced 1 d and 3d after the MNU administration compared to those of the N group (all *P* < 0.01). However, there were no statistical differences in the nuclei counts of ONL between the M group and the R group at both time points (all *P* > 0.05) (Figure 3).

### The effect of RSV on photoreceptor apoptosis

**Figure 4.**
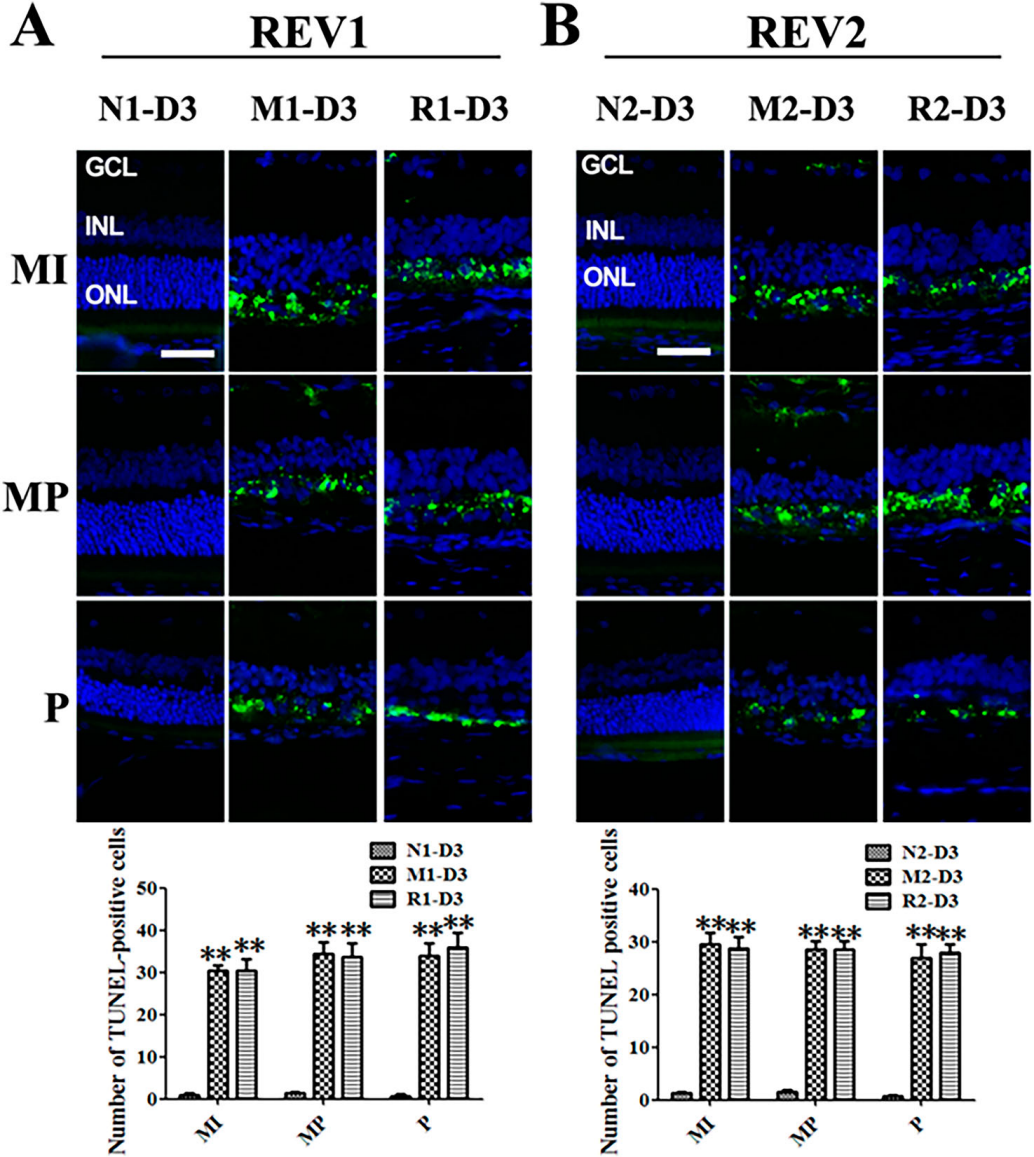
RSV-induced effect on cellular apoptosis in MNU-damaged retinas. A: Representative images of TUNEL assay and plot of quantification of TUNEL-positive cells in retinal sections from rats treated with RSV intervention of the first mode. B: Representative images of TUNEL assay and plot of quantification of TUNEL-positive cells in retinal sections from rats treated with RSV intervention of the second mode. (MI: the middle area of the retina; MP: the mid-peripheral area of the retina; P: the peripheral area of the retina; ONL: outer nuclear layer; INL: inner nuclear layer; GCL: ganglion cell layer; Scale bar: 50 µm; **P < 0.01 vs. vehicle group).

At 3 d after the MNU administration, the TUNEL-positive cells emerged obviously in the ONL from the middle retina to the peripheral regions in the M group compared to the N group. The rat retinas from the R group also showed the obvious sign of TUNEL-positive cells from the middleretina to the peripheral regions. The numbers of TUNEL-positive cells in the M group and the R group were not significantly different (all *P* > 0.05) (Figure 4).

### The effect of RSV on microglia activation of MNU-treated retinas

**Figure 5.**
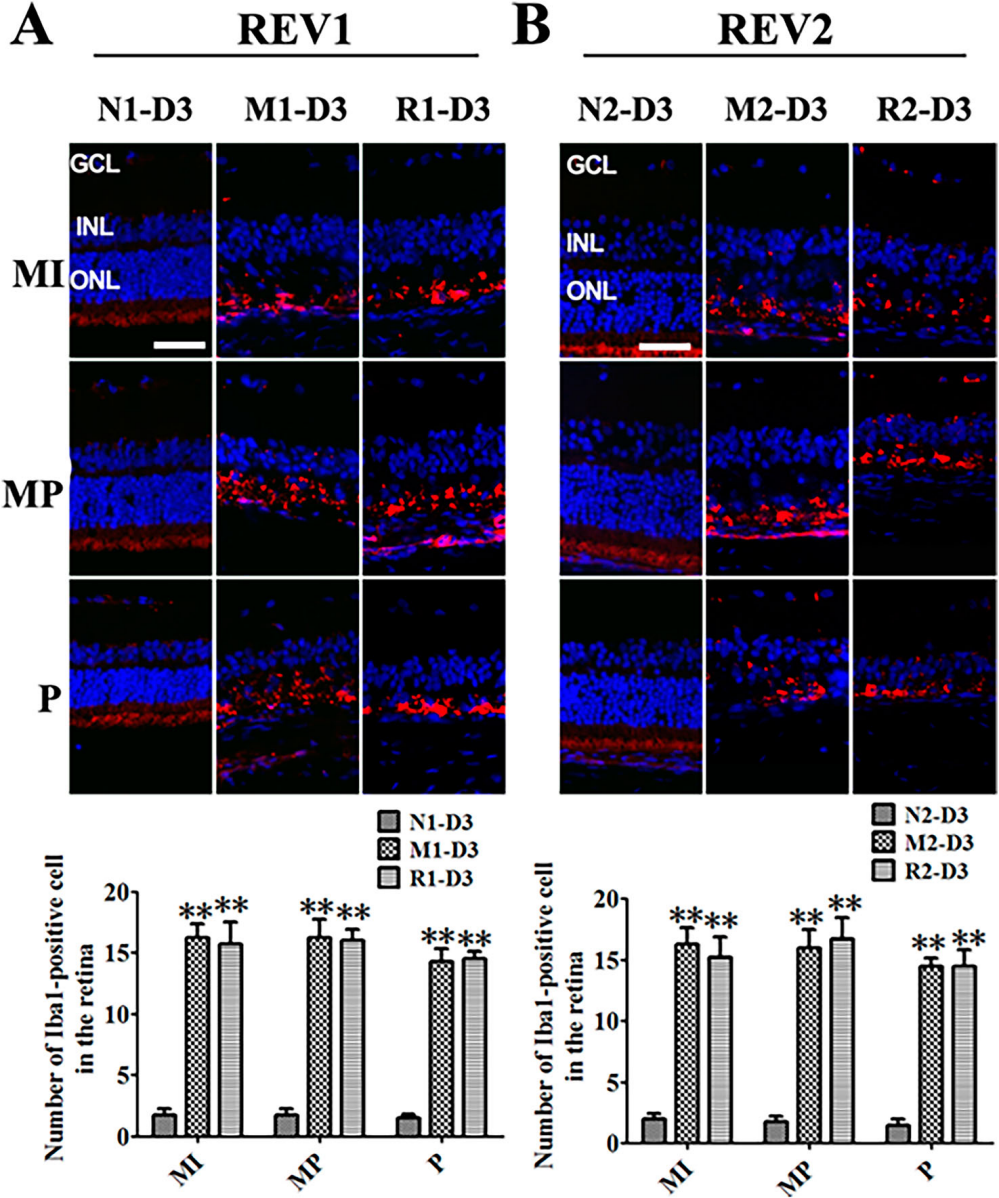
RSV-induced effect on microglia activation in MNU-treated retinas. A: Representative photomicrographs of Iba1 immunofluorescence in the retina and quantification of Iba1-positive cells in the retinas with RSV intervention of the first mode. B: Representative photomicrographs of Iba1 immunofluorescence in the retina and quantification of Iba1-positive cells in the retinas with RSV intervention of the second mode. (*n* = 3; MI: the middle area of the retina; MP: the mid-peripheral area of the retina; P: the peripheral area of the retina; ONL: outer nuclear layer; INL: inner nuclear layer; GCL: ganglion cell layer; Scale bar: 50 µm; **P < 0.01 vs. vehicle group).

The Iba1-positive cells were obviously found in the outer retina surrounding the remaining photoreceptors from the middle to peripheral regions in the rat retinas of the M and the R groups at 3 d after MNU administration. However, the number of Iba1-positive cells from the R group was not statistically different from that of the M group (all *P *> 0.05) (Figure 5).

### The RSV-induced effect on SIRT1 expression in MNU-treated retinas

**Figure 6.**
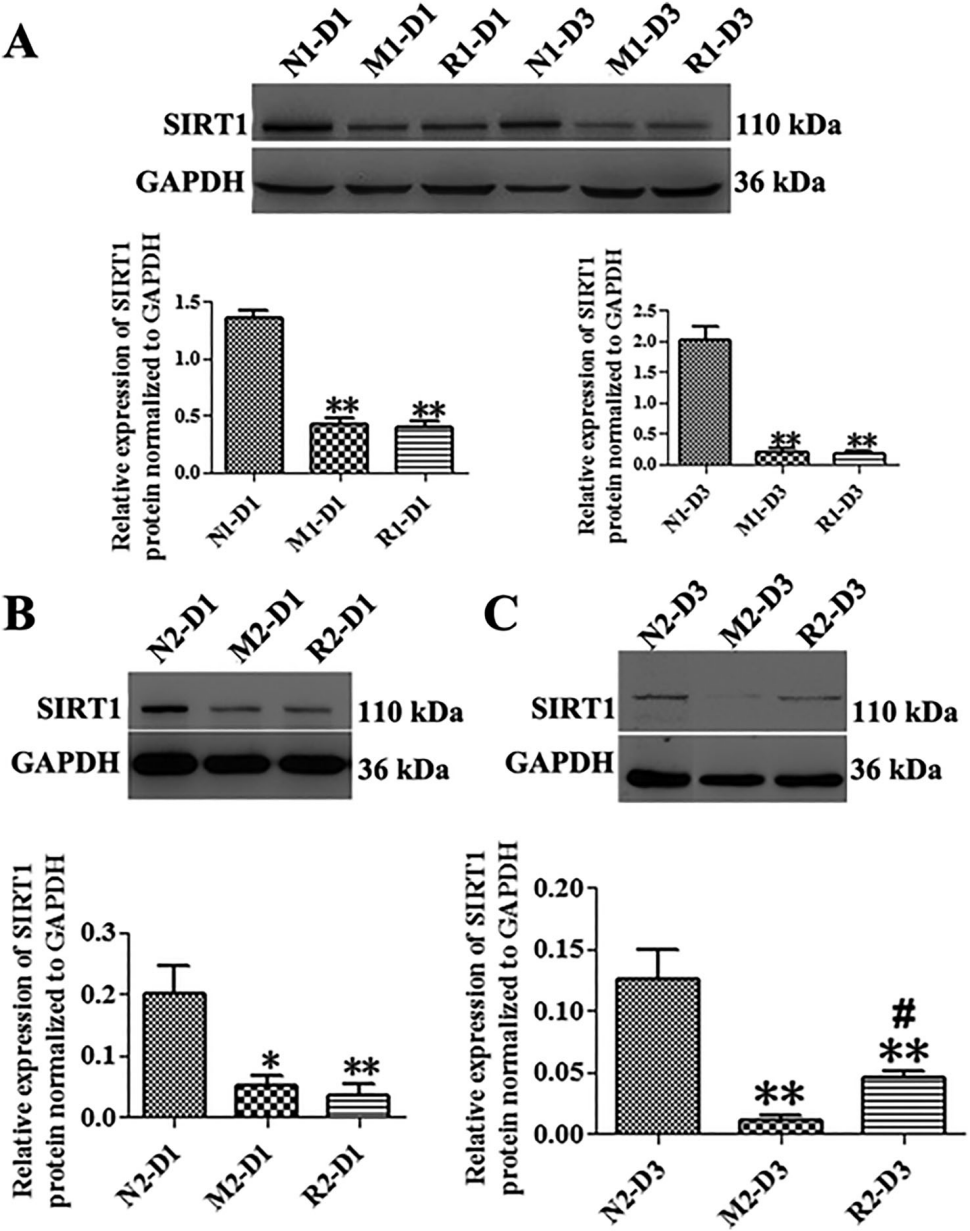
RSV-induced effect on SIRT1 protein expression in MNU-treated retinas. A: Representative Western blot bands and quantitative analysis of SIRT1 protein expression of retinas with RSV intervention of the first mode; B, C: Representative Western blot bands and quantitative analysis of SIRT1 protein expression of retinas with RSV intervention of the second mode at 1 d (B) and 3 d (C) after MNU administration. (*n* = 3; *P < 0.05, **P < 0.01: vs. N group; ^#^*P* < 0.05: vs. M group).

The MNU significantly decreased the expression of SIRT1 protein in the M group in comparison with that of the N group at both 1 and 3 d, (the N group vs. M group: 2.03 ± 0.21 *vs.* 0.20 ± 0.06 fold for 1 d; 0.13 ± 0.02 *vs*. 0.01 ± 0.01 fold for 3d, all *P* < 0.01). The expression of SIRT1 protein of the rat retinas in the R group of the first mode did not show any statistical differences from that of the M group at both time points (all *P *> 0.05). The SIRT1 protein in the retinas in the R group of the second mode was not statistically increased from that of the M group at 1 d (*P* > 0.05), while it was slightly increased at 3 d compared to that of the M group (*P* < 0.05) (Figure 6).

## Discussion

In our study, various techniques were performed to evaluate the impacts of RSV on the MNU-induced RP rats expecting to find out the relief on the retinal degeneration. RSV was administered in the two modes, with the first one served as the treatment mode and the second served as the prevention. The treatment mode was given at a large dose, which was reported previously (Kubota et al. 2009) and was within the tolerated dose of rats (Williams et al. 2009). Meanwhile, the dose of prevention mode was applied as it was confirmed safe and effective in a previous study (Qi et al. 2015).

MNU, an alkylating agent, was applied to induce the retinal degeneration in rat, which was a commonly-used RP model (Yan et al. 2020). The alkylation of MNU with DNA could produce the 7-methyldeoxyguanosine-DNA adduct insides the photoreceptor nuclei, leading to the selective apoptosis of photoreceptors in rats (Nakajima et al. 1996). The high density of ONL in our OCT image was deemed to be caused by the shrinkage of the apoptotic photoreceptors, which reflected more light from the cell surface and increased the optical attenuation coefficient (van der Meer et al. 2010). This phenomenon was supported by our TUNEL data, which showed that multiple apoptotic cells emerged in the ONL. Similarly, apoptotic photoreceptors with changes in the scattering were monitored in real-time OCT and were attributed to the intracellular morphological changes in other studies and our previous research (de Bruin et al. 2016; Yan et al. 2020). However, the RSV intervention in our study could not reverse or ameliorate the above process. Besides, the retinal morphological degeneration was not obviously alleviated according to the statistical analysis of ONL nuclei counts and the TUNEL-positive cells number.

The deterioration of retinal function in rats could be induced by the MNU administration, in addition to the retinal morphological changes (Chen et al. 2016). In our study, the ERG waveforms became undistinguished and the retinal ONL was almost completely lost out in three days after the MNU administration. The ERG amplitudes in the dark-adapted condition from the MNU-treated rats were only approximately 5–10% of the rats from the normal control group, while the amplitudes in the light-adapted condition were about 30–60% of the normal control group. This phenomenon was supported by other studies, which argued that the rods were instinctively more vulnerable to the toxicity of MNU than the cones (Tao et al. 2015). RSV, which could rescue the retinal function in light-damaged retinas, failed to preserve the decrease of ERG amplitudes in both the dark-adapted and light-adapted conditions in the MNU-treated rats in our study.

Microglia cell activation was extensively established as an indicator associated with the retinal degenerative diseases, included RP (Langmann 2007). Microglia cell activation in the retina was primarily caused by the inflammatory media released from the apoptotic photoreceptors debris and the loss of inhibitory input from the surrounding cells (Dick et al. 2003). The activated microglia cells would help to clear up the debris. However, they could also cause excessive activation of other microglia cells and kill the adjacent photoreceptors during the consistent pathological status (Karlstetter et al. 2015). Several medicine that could decrease the microglia cells activation were shown effective in ameliorating RP in rodent models (Wang et al. 2017). RSV was found to provide the neuroprotection by counteracting the excessive microglia cell activation in the neuroinflammation models (Yang et al. 2017). Our result of Iba1 immunofluorescence staining verified the involvement of microglia cell activation in MNU-induced RP rats which was in accordance with other RP models (Noailles et al. 2016). However, our data found that the RSV intervention could not alleviate the microglia cell activation in the RP rat retinas.

The decreased expression of SIRT1, the NAD^+^-dependent deacetylase, was detected in the MNU-induced RP rats of our study. SIRT1 expression was also discovered to be decreased in MNU-induced mammary tumors (Fang et al. 2015). RSV, the widely-used SIRT1-activating compound, was showed to protect the LPS/IFNγ-treated N9 microglia cells via upregulation of SIRT1 (Zhang et al. 2017). However, our study found that the SIRT1 protein could not be upregulated by RSV in the treatment mode, and the prevention mode of 1 d. The SIRT1 expression was somewhat elevated on the 3 d of the prevention mode. However, the slight upregulation of SIRT1 could not make a significant difference in the outcome of retinal degeneration in the MNU-induced RP rats.

SIRT1 was found to depend on the NAD^+^ to catalyze the deacetylation of specific amino-acetylated lysine residues of the targeted protein substrates to obtain the biological effects (Pacholec et al. 2010). NAD^+^ from the MNU-induced RP model was largely consumed out as a substrate of the poly (ADP-ribose) polymerase (PARP), which was activated for repairing the MNU-damaged DNA (Uehara et al. 2006). The loss of photoreceptor cells during the MNU-induced RP was suppressed with an injection of nicotinamide, an NAD^+^ precursor. Specifically, the nicotinamide was showed to ameliorate the loss of cellular NAD^+^, which prevented ATP deficiency, energy loss, and subsequent cell death (Uehara et al. 2006). Even though RSV could upregulate the SIRT1 expression, there would be little protective effect due to the primary severe loss of NAD^+^ in the MNU-induced RP.

In conclusion, our data suggest that RSV was unable to ameliorate the MNU-induced photoreceptor degeneration in rat retina. The underlying mechanisms might be attributed to the MNU-induced depletion of NAD^+^, which precluded the deacetylation of SIRT1 and the following beneficial effects.
